# Breakthroughs in Medicinal Chemistry: New Targets and Mechanisms, New Drugs, New Hopes–6

**DOI:** 10.3390/molecules25010119

**Published:** 2019-12-28

**Authors:** Jean Jacques Vanden Eynde, Arduino A. Mangoni, Jarkko Rautio, Jérôme Leprince, Yasu-Taka Azuma, Alfonso T. García-Sosa, Christopher Hulme, Josef Jampilek, Rafik Karaman, Wei Li, Paula A. C. Gomes, Dimitra Hadjipavlou-Litina, Raffaele Capasso, Athina Geronikaki, Laura Cerchia, Jean-Marc Sabatier, Rino Ragno, Tiziano Tuccinardi, Andrea Trabocchi, Jean-Yves Winum, F. Javier Luque, Katalin Prokai-Tatrai, Mariana Spetea, Michael Gütschow, Ivan Kosalec, Catherine Guillou, M. Helena Vasconcelos, George Kokotos, Giulio Rastelli, Maria Emília de Sousa, Clementina Manera, Sandra Gemma, Stefano Mangani, Carlo Siciliano, Stefania Galdiero, Hong Liu, Peter J. H. Scott, Cristóbal de los Ríos, Luigi A. Agrofoglio, Simona Collina, Rita C. Guedes, Diego Muñoz-Torrero

**Affiliations:** 1 Formerly head of the Department of Organic Chemistry (FS), University of Mons-UMONS, 7000 Mons, Belgium; Jean-Jacques.VANDENEYNDE@ex.umons.ac.be; 2 Discipline of Clinical Pharmacology, College of Medicine and Public Health, Flinders University and Flinders Medical Centre, Bedford Park 5042, Adelaide, Australia; arduino.mangoni@flinders.edu.au; 3 Medizinische Fakultät Carl Gustav Carus, Technische Universität Dresden, 01069 Dresden, Germany; 4 School of Pharmacy, Faculty of Health Sciences, University of Eastern Finland, P.O. Box 1627, FI-70211 Kuopio, Finland; jarkko.t.rautio@uef.fi; 5 UNIROUEN, Inserm U1239, Laboratory of Neuronal and Neuroendocrine Communication and Differentiation, Normandie University, 76000 Rouen, France; jerome.leprince@univ-rouen.fr; 6 UNIROUEN, Regional Cell Imaging Platform of Normandy (PRIMACEN), Normandie University, 76000 Rouen, France; 7 Laboratory of Veterinary Pharmacology, Division of Veterinary Science, Osaka Prefecture University Graduate School of Life and Environmental Sciences, 1-58 Rinku-ohraikita, Izumisano, Osaka 598-8531, Japan; azuma@vet.osakafu-u.ac.jp; 8 Institute of Chemistry, University of Tartu, Ravila 14a, 50411 Tartu, Estonia; alfonso.tlatoani.garcia.sosa@ut.ee; 9 Department of Pharmacology and Toxicology, and Department of Chemistry and Biochemistry, College of Pharmacy, The University of Arizona, Biological Sciences West Room 351, 1041 East Lowell Street, Tucson, AZ 85721, USA; hulme@pharmacy.arizona.edu; 10 Department of Analytical Chemistry, Faculty of Natural Sciences, Comenius University, Ilkovicova 6, 842 15 Bratislava, Slovakia; josef.jampilek@gmail.com; 11 Pharmaceutical & Medicinal Chemistry Department, Faculty of Pharmacy, Al-Quds University, Jerusalem P.O. Box 20002, Palestine; dr_karaman@yahoo.com; 12 Department of Sciences, University of Basilicata, Viadell’Ateneo Lucano 10, 85100 Potenza, Italy; 13 Department of Pharmaceutical Sciences, College of Pharmacy, University of Tennessee Health Science Center, Memphis, TN 38163, USA; wli@uthsc.edu; 14 LAQV-REQUIMTE, Departamento de Química e Bioquímica, Faculdade de Ciências da Universidade do Porto, Rua do Campo Alegre 687, 4169-007 Porto, Portugal; pgomes@fc.up.pt; 15 Department of Pharmaceutical Chemistry, School of Pharmacy, Faculty of Health Sciences, Aristotle University of Thessaloniki, 54124 Thessaloniki, Greece; hadjipav@pharm.auth.gr (D.H.-L.)geronik@pharm.auth.gr (A.G.); 16 Department of Agricultural Sciences, University of Naples Federico II, Via Università 100, 80055 Portici (NA), Italy; rafcapas@unina.it; 17 Institute of Experimental Endocrinology and Oncology “G. Salvatore” (IEOS), National Research Council (CNR), 80131 Naples, Italy; cerchia@unina.it; 18 Institute of NeuroPhysiopathology, UMR 7051, Faculté de Médecine Secteur Nord, 51, Boulevard Pierre Dramard-CS80011, 13344-Marseille CEDEX 15, France; sabatier.jm1@libertysurf.fr; 19 Rome Center for Molecular Design, Department of Drug Chemistry and Technology, Sapienza University, P.le Aldo Moro 5, 00185 Rome, Italy; rino.ragno@uniroma1.it; 20 Department of Pharmacy, University of Pisa, Via Bonanno 6, 56126 Pisa, Italy; tiziano.tuccinardi@unipi.it (T.T.); clementina.manera@farm.unipi.it (C.M.); 21 Department of Chemistry “Ugo Schiff”, University of Florence, via della Lastruccia 13, I-50019 Sesto Fiorentino, Florence, Italy; andrea.trabocchi@unifi.it; 22 Institut des Biomolécules Max Mousseron (IBMM), École nationale supérieure de chimie de Montpellier (ENSCM), Université de Montpellier, CEDEX 05, 34296 Montpellier, France; jean-yves.winum@umontpellier.fr; 23 Department of Nutrition, Food Sciences and Gastronomy, Faculty of Pharmacy and Food Sciences, Institute of Biomedicine (IBUB) and Institute of Theoretical and Computational Chemistry (IQTC), University of Barcelona, Av. Prat de la Riba 171, E-08921 Santa Coloma de Gramenet, Spain; fjluque@ub.edu; 24 Department of Pharmacology and Neuroscience, University of North Texas Health Science Center, 3500 Camp Bowie Blvd, Fort Worth, TX 76107, USA; Katalin.Prokai@unthsc.edu; 25 Department of Pharmaceutical Chemistry, Institute of Pharmacy and Center for Molecular Biosciences Innsbruck (CMBI), University of Innsbruck, 6020 Innsbruck, Austria; mariana.spetea@uibk.ac.at; 26 Pharmaceutical Institute, Pharmaceutical & Medicinal Chemistry, University of Bonn, An der Immenburg 4, 53115 Bonn, Germany; guetschow@uni-bonn.de; 27 Faculty of Pharmacy and Biochemistry, University of Zagreb, A. Kovačića 1, HR-10000 Zagreb, Croatia; ikosalec@pharma.hr; 28 Institut de Chimie des Substances Naturelles, CNRS UPR 2301, Unversité de Paris-Saclay, 1 av. de la Terrasse, 91198 Gif-sur-Yvette, France; catherine.guillou@cnrs.fr; 29 i3S-Instituto de Investigação e Inovação em Saúde, Universidade do Porto, Rua Alfredo Allen 208, 4200-135 Porto, Portugal; hvasconcelos@ipatimup.pt; 30 Cancer Drug Resistance Group-IPATIMUP-Institute of Molecular Pathology and Immunology of the University of Porto, Rua Júlio Amaral de Carvalho, 45, 4200-135 Porto, Portugal; 31 Department of Biological Sciences, FFUP-Faculty of Pharmacy, University of Porto, Rua de Jorge Viterbo Ferreira 228, 4050-313 Porto, Portugal; 32 Department of Chemistry, National and Kapodistrian University of Athens, Panepistimiopolis, 15771 Athens, Greece; gkokotos@chem.uoa.gr; 33 Department of Life Sciences, University of Modena and Reggio Emilia, Via Giuseppe Campi 103, 41125 Modena, Italy; giulio.rastelli@unimore.it; 34 Laboratório de Química Orgânica e Farmacêutica, Departamento de Ciências, Químicas, Faculdade de Farmácia, Universidade do Porto, Rua Jorge Viterbo Ferreira 228, 4050-313 Porto, Portugal; esousa@ff.up.pt; 35 Interdisciplinar de Investigação Marinha e Ambiental (CIIMAR/CIMAR), Universidade do Porto, Terminal de Cruzeiros do Porto de Leixões, Avenida General Norton de Matos, S/N 4450-208 Matosinhos, Portugal; 36 Department of Biotechnology, Chemistry and Pharmacy, DoE 2018-2022, University of Siena, via Aldo Moro 2, 53100 Siena, Italy; GEMMA@UNISI.IT (S.G.); stefano.mangani@unisi.it (S.M.); 37 Department of Pharmacy, Health and Nutritional Sciences, University of Calabria, I-87036 Arcavacata di Rende, Italy; carlo.siciliano@unical.it; 38 Department of Pharmacy, University of Naples Federico II, Via Mezzocannone 16, 80134 Naples, Italy; stefania.galdiero@unina.it; 39 State Key Laboratory of Drug Research, Shanghai Institute of Materia Medica, Chinese Academy of Sciences, 555 Zu Chong Zhi Road, Shanghai 201203, China; hliu@simm.ac.cn; 40 Department of Radiology, University of Michigan, Ann Arbor, MI 48105, USA; pjhscott@med.umich.edu; 41 Instituto de Investigación Sanitaria, Servicio de Farmacología Clínica, Hospital Universitario de la Princesa, 28006 Madrid, Spain; cristobal.delosrios@inv.uam.es; 42 ICOA, CNRS UMR 7311, Université d’Orleans, Rue de Chartres, 45067 Orleans CEDEX 2, France; luigi.agrofoglio@univ-orleans.fr; 43 Department of Drug Sciences, Medicinal Chemistry and Pharmaceutical Technology Section, University of Pavia, Viale Taramelli 12, 27100 Pavia, Italy; simona.collina@unipv.it; 44 iMed.Ulisboa and Faculdade de Farmácia, Universidade de Lisboa, 1649-003 Lisbon, Portugal; rguedes@ff.ulisboa.pt; 45 Laboratory of Pharmaceutical Chemistry, Faculty of Pharmacy and Food Sciences, and Institute of Biomedicine (IBUB), University of Barcelona, Av. Joan XXIII, 27-31, E-08028 Barcelona, Spain

## 1. Introduction

Breakthroughs in Medicinal Chemistry: New Targets and Mechanisms, New Drugs, New Hopes is a series of Editorials that is published on a biannual basis by the Editorial Board of the Medicinal Chemistry section of the journal *Molecules*. In these Editorials, we highlight in brief reports (of about one hundred words) a number of recently published articles that describe crucial findings, such as the discovery of novel drug targets and mechanisms of action or novel classes of drugs, which may inspire future medicinal chemistry endeavors devoted to addressing prime unmet medical needs.

## 2. Siderophores, Did You Say Siderophores?

### *Highlighted by Jean Jacques Vanden Eynde* 

**Figure 1:**
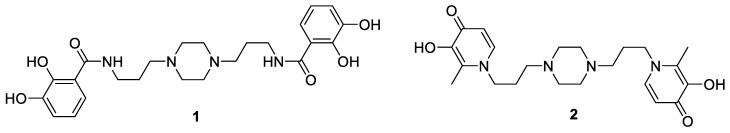
Chemical structure of siderophore mimetics.

Ferric ions (Fe^3 +^) play a vital role in many cellular processes but their poor solubility under biological conditions hampers their transmembrane movements. The situation is facilitated by chelating the ion, and many microorganisms are capable of secreting such chelates, which are called siderophores (from the ancient Greek language: “iron carrier”). Designing siderophore mimetics (Figure 1) is expected to combat bacteria and fungi by introducing either toxic metals or antibiotics into the organism. This strategy has been recently exploited by the team of P. Sonnet [1] in order to evaluate the antipseudomonal activities and cytotoxicity of conjugates bearing a piperazine moiety and a catechol (**1**) or an hydroxypyridone (**2**) chelating group.

## 3. A Combined Neuroprotective Approach for Dementia

### *Highlighted by Arduino A. Mangoni* 

The identification of new, effective drugs for the treatment of dementia remains challenging. Targeting multiple pathways involved in the onset and the progression of the disease might increase the success rate of drug discovery and development programs [2]. Scheiner et al. report the synthesis of a series of hybrid molecules that are based on the cholinesterase inhibitor tacrine and a benzimidazole-based human cannabinoid receptor subtype 2 agonist [3]. The hybrids exhibited significant inhibitory effects towards cholinesterase activity and Aβ40 and Aβ42 aggregation, and concomitant partial agonist activity towards the human cannabinoid receptor subtype 2. Furthermore, the compound 2-(4-ethoxybenzyl)-1-isopentyl-*N*-(2-((2-((1,2,3,4-tetrahydroacridin-9-yl)amino)ethyl)disulfaneyl)ethyl)-1*H*-benzo[*d*]imidazole-5-carboxamide exerted significant, concentration-dependent, neuroprotective effects in murine hippocampal neurons and prevented Aβ25-35-induced short- and long-term memory impairment in mice [3]. Notably, animal studies did not show any evidence of hepatotoxicity, a common complication during treatment with tacrine [4]. Pending further in vivo investigations, the results of this study suggest that agents with dual cholinesterase inhibition and human cannabinoid receptor subtype 2 activation might represent a promising therapeutic strategy for dementia.

## 4. Prodrug Strategy for Multiple Charged Drugs

### *Highlighted by Jarkko Rautio* 

2-(Phosphonomethyl)pentanedioic acid (2-PMPA), which is a potent (IC_50_ = 300 pM) and selective inhibitor of glutamate carboxypeptidase II, has demonstrated preclinical efficacy in various neurological and psychiatric disease models as well as in inflammatory bowel disease and cancer models. However, due to its four acidic functionalities that are ionized at physiological pH it suffers from poor oral bioavailability (<1%) and has not been clinically developed. Masking two to four of the acidic groups using (5-methyl-2-oxo-1,3-dioxol-4-yl)methyl (ODOL), a cyclic carbonate promoiety significantly improved lipophilicity (clogP) compared to that of 2-PMPA. All prodrugs delivered significantly higher 2-PMPA plasma concentrations in a single time-point screening study in mice. The best prodrug consisting of four ODOL promoieties demonstrated an 80-fold enhancement in exposure versus oral 2-PMPA in an extended time-course pharmacokinetic study in mice with an absolute oral bioavailability of 50%. This prodrug also delivered a 44-fold enhanced 2-PMPA plasma exposure in further studies in dogs. Results demonstrate the applicability of the FDA approved ODOL promoiety in multiple charged drugs [5].

## 5. A SOX9-Derived Peptide Inhibits the Growth of Colorectal Cancer Tumors

### *Highlighted by Jérôme Leprince* 

The oncogenic Wnt/β-catenin signaling pathway is constitutively activated in all inherited, and in 80% sporadic, colorectal cancers. This activation results in an accumulation of non-phosphorylated β-catenin in the nucleus of tumor cells, which activates the T-cell transcription factor leading to the expression of pro-oncogenic Wnt/β-catenin signaling target genes, such as c-Myc. Recently, Blache et al. reported that doxycycline-evoked expression of S9pep, a 21-residue long fragment of the transcription factor SOX9, inhibits Wnt/β-catenin signaling and c-Myc expression by β-catenin relocation into the cytoplasm [6]. A syngenic graft of S9pep-expressing colorectal cancer cells in mice reduces tumor growth in comparison to non-expressing tumor cells. These results pave the way toward the development of promising S9pep analogues to tackle colorectal cancers.

## 6. Ustekinumab, an Anti-IL-12/23 p40 Monoclonal Antibody, as a Molecularly-Targeted Therapy for the Treatment of Crohn’s Disease

### *Highlighted by Yasu-Taka Azuma* 

Molecularly-targeted therapy is a therapy that treats a specific molecule by targeting it and controlling its function. IL-12 is a heterodimer consisting of the p40 and p35 subunits. On the other hand, IL-23 is a heterodimer in which the p19 subunit is bound to the same p40 subunit as IL-12. IL-12 induces Th1 cell differentiation, whereas IL-23 induces Th17 cell differentiation. By suppressing p40, a common subunit, it is possible to suppress both Th1 and Th17. In particular, p40 is taken up as a therapeutic target molecule for Crohn’s disease [7]. Li et al. reported the effect of ustekinumab, a monoclonal antibody against p40, on the microscopic manifestations of Crohn’s disease [8]. Improvement was observed 8 weeks after administration of this antibody. Thereafter, observation was made up to the 44th week. Improvement was observed even at 12 week intervals, and further improvement was observed at 8 week intervals over the 12 week intervals. This antibody has further expanded the options for treating Crohn’s disease.

## 7. A Stab in the Back of TB: Recovering *Mycobacterium tuberculosis* Fumarate Hydratase Through a Non-Conserved Allosteric Site

### *Highlighted by Alfonso T. García-Sosa* 

Despite a long history, an estimated fourth of the world’s population is infected with *M. tuberculosis*. It kills over one million people each year and resistance to current therapeutics is becoming widely spread and transmissible. The bacteria’s fumarate hydratase enzyme is highly conserved with the human homologue. Promising work [9] succeeded in targeting a non-conserved allosteric site on the highly-conserved (with conserved active site) enzyme, which makes the compounds all the more interesting due to the possibility of a higher selectivity index against the human enzyme, as well as being able to permeate the mycobacterial cell wall. 

Whitehouse et al. [9] recovered a previous lead compound with a unique dimeric, induced-fit binding mode and MIC of 250 μM by SAR on head and tail functional groups to achieve new compounds with activity against both fumarate hydratase and *M. tuberculosis* H37Rv bacteria. 

The compound with the best MIC value was *N*-(5-(azepan-1-ylsulfonyl)-2-methoxyphenyl)-2- (benzofuran-3-yl)acetamide; and that with the best IC_50_ value was *N*-(5-(azocan-1-ylsulfonyl)-2-methoxyphenyl)-2-(4-oxo-3,4-dihydrophthalazin-1-yl)acetamide, thus rescuing both a target and a compound series.

## 8. New Hopes for 3rd Generation Castration Resistant Prostate Cancer Drugs: Arv7 Splice Variant Binders

### *Highlighted by Christopher Hulme* 

Androgen receptor (AR) reactivation is thought to be the key mechanism that drives castration-resistant prostate cancer (CRPC) resistance to second generation antiandrogens, like enzalutamide, which partly involves the generation of AR splice variants lacking a ligand binding domain (LBD). As such, a dominant splice variant is Arv7, and high content screening, followed by optimization delivered the small molecule JJ-450, (4-(5-chloro-2-methylphenyl)piperazin-1-yl) ((1*RS*,2*SR*)-2-(4-fluorophenyl)cyclopropyl)methanone, which inhibits Arv7 transcriptional activity and its target gene expression. Moreover, the molecule inhibits the growth of CRPC tumor xenografts, including ARv7-expressing 22Rv1. Taken together, the results are highly suggestive that JJ-450 is a new class of AR antagonist with potential as a third generation therapeutic for CRPC, which critically is effective in animals resistant to the LBD binder enzalutamide [10].

## 9. Multi-Target Drugs for Rheumatoid Arthritis Management

### *Highlighted by Josef Jampilek* 

Rheumatoid arthritis (RA) is a systemic inflammatory autoimmune disease leading to chronic inflammation of synovial tissue with subsequent disintegration of articular cartilage and bone. Common symptoms are joint pain, stiffness, swelling, and deformities. Inflammation leads to respiratory and cardiovascular diseases increasing RA mortality. Pharmacological treatment to alleviate inflammation and reduce the progression of structural damage frequently consists of the administration of nonsteroidal anti-inflammatory drugs (NSAIDs), glucocorticoids, methotrexate, and biological drugs. Despite the progress in treatment, patients still do not have a sufficient response to therapy or suffer severe side effects [11]. The multi-target approach in drug design leads to the preparation of hybrids of NSAIDs and carbon anhydrase inhibitors (CAIs) [12,13]. Supuran’s research team designed molecules formed by an ester bridge linking NSAIDs with coumarin type CAI. Adducts of 6-/7-hydroxycoumarins with ketoprofen inhibited CA IV, IX, XII isoforms in the nanomolar range and COX-1/COX-2. (±)-2-[(2-Oxo-2*H*-chromen-6-yl)oxy]ethyl 2-(3-benzoylphenyl)propanoate demonstrated the most promising antihyperalgesic effect in the in vivo rat RA model. These NSAID-CAI hybrids are cleaved in plasma depending on the NSAID used but are easier than their pattern amide isosteres [14].

## 10. Sodium Oligomannate—A Novel Drug for the Treatment of Mild to Moderate Alzheimer’s Disease

### *Highlighted by Rafik Karaman* 

Dementia is characterized by a decline in memory, language and other thinking skills that affect the ability of a person to execute daily activities. According to WHO, the number of people affected by dementia is more than 50 million worldwide, Alzheimer’s disease (AD) is the most common form of dementia and accounts for 50 to 60% of cases. Therefore, drugs having the ability to slow down or prevent all forms of dementia are crucially needed.

In a 9-month phase 3 clinical trial involving 818 patients, the drug oligomannate showed improved cognitive function after just 4 weeks in patients with mild to moderate AD. Oligomannate was not found to target β-amyloid proteins in the brain but instead, it alters the connection between the microbiome and the brain. Based on the results that oligomannate suppresses gut dysbiosis and phenylalanine/isoleucine accumulation, harnesses neuroinflammation and reverses cognition impairment, it was concluded that oligomannate has the potential to inhibit the progression of AD. Researchers are now convinced that gut bacteria may be involved in many neurological disorders including AD. The oligomannate discovery opens the door widely to address many other diseases via targeting the microbiome [15].

## 11. New Strategies to Reduce Off-Target Toxicity and Identify True Targets to Improve Clinical Successes

### *Highlighted by Wei Li* 

The chance of a new drug progressing from its entrance in clinical trials to FDA approval is only 3%. The most common causes for failures are inadequate efficacy and/or dose-limiting toxicities, but the lack of more rigorous validation of the drug target plays a significant role, as demonstrated by a recent study [16]. The authors analyzed several drugs and drug targets that are currently in clinical trials and found that some targets are actually non-essential for cancer cell growth, and that the anticancer effects for certain drugs actually result from unintended off-target effects. The key to these studies is to use more precise CRISPR-Cas9 techniques instead of traditional RNA interference or small-molecule inhibitors. They further demonstrated that the true target for the drug OTS964 is CDK11, not the reported PBK. Given the importance of identifying reliable biomarkers for patient selection, a more stringent genetic validation of the drug target in preclinical settings is very critical to decrease the high failure rate in drug development programs and significantly benefit both drug developers and cancer patients.

## 12. Crossing a Formidable Barrier: Seven Molecular Descriptors for BBB Permeability Prediction

### *Highlighted by Paula A. C. Gomes* 

After the advent of combinatorial synthesis and high-throughput screening for fast selection of vast numbers of hits, it was perceived that many such hits did not possess adequate pharmacokinetics. This led to the “drug-likeness” concept, where physicochemical descriptors are used to predict how “druggable” a compound is, pioneered by Lipinsky with his “Rule of Five” proposed in 1997 [17]. Since then, diverse algorithms have been advanced to estimate the bioavailability of a compound [18], but tools to predict the ability to cross the blood–brain barrier (BBB) remain scarce. Anticipation of such ability is crucial for any drug candidate, as while some drugs have to cross the BBB to exert their action, others should not, to avoid unwanted effects. Hence, the “BBB Score”, recently proposed by Weaver and co-workers [19] is highly relevant for medicinal chemists. The “BBB score” introduces an algorithm to estimate the BBB permeation ability based on molecular weight, topological polar surface area, p*K*a, and the number of aromatic rings, heavy atoms, hydrogen bond donors, and hydrogen bond acceptors, and is a valuable add-on to current tools for drug design.

## 13. Design, Synthesis and Biological Evaluation of Second-Generation NLRP3 Inflammasome Inhibitors

### *Highlighted by Dimitra Hadjipavlou-Litina* 

**Figure 2:**
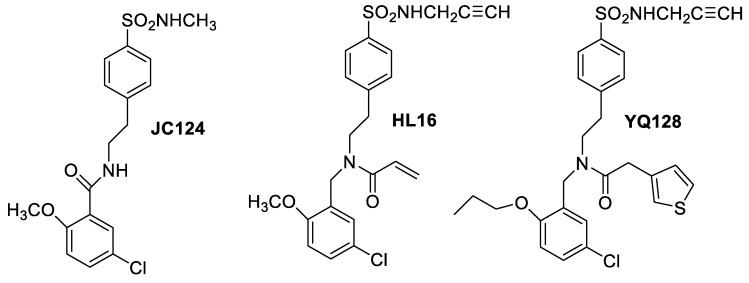
Modification of JC124 used for the design of the new scaffold HL16 that led to the new selective lead YQ128.

Inflammasomes are multimeric protein complexes that assemble in the cytosol after sensing pathogen-associated molecular patterns (PAMPs) or danger-associated molecular patterns (DAMPs) and are linked to a variety of autoinflammatory and autoimmune diseases, including neurodegenerative diseases and metabolic disorders. Thus, NLRP3 inflammasomes have been recently described as an attractive drug target for neurodegenerative disorders. Several small-molecule inhibitors have been recently reported to block the NLRP3 inflammasome pathways. Zhang et al. [20] suggested earlier that sulfonamide analogues like JC124 can directly interfere with the formation of the NLRP3 inflammasome complex. Further development of analogues based on this template with improved potency and drug-like properties was presented, with the design of HL16 [21]. However, HL16 showed no selectivity. Improvement of the structure (Figure 2) with replacement of the 2-OCH_3_ group and the presence of a heteroaromatic acetamide provided analogues with improved potency, leading to a new selective lead, YQ128, with an IC_50_ of 0.30 μM. In vivo models confirmed its brain penetration. Pharmacokinetic studies at 20 mg/kg in rats indicated extensive systemic clearance, tissue distribution with a half-life of 6.6 h, low oral bioavailability and no efflux transport.

## 14. Sphingosine-1-Phosphate Signaling Is Involved in Cigarette Smoke-Induced Airway Pathology

### *Highlighted by Raffaele Capasso* 

The most important risk factor for COPD is cigarette smoking. COPD is characterized by progressive obstruction of airflow, not fully reversible, caused by deleterious particles or gases in lung parenchyma. There are currently no specific COPD treatments and smoking cessation remains the most effective therapeutic intervention. Recently De Cunto et al. demonstrated the involvement of sphingosine-1-phosphate (S1P) signalling in the progression of airway dysfunction associated with emphysematous lesions following chronic exposure to cigarette smoke [22]. In their experimental setting cigarette smoke triggers a lung inflammation [23], that slowly develops and promotes the airway structural changes responsible for the cholinergic hyper-responsiveness of both lower (early response) and upper (later response) airways from month 9 onwards. The authors support the hypothesis of an active role of Sph-K/S1P signalling pathway in the altered cholinergic control of airway function [24] triggered by cigarette smoke. Thus, the S1P pathway represents a feasible therapeutic target in airway hyperresponsiveness associated with emphysema and, particularly in smokers, with both asthma and COPD (ACOS patients), a patient category more exposed to the disease progression and mortality.

## 15. New Class of Antifungals against *Aspergillus fumigatus*

### *Highlighted by Athina Geronikaki* 

In the last decades, fungal infections have been a growing problem worldwide causing morbidity and mortality. Their treatment still remains an important and challenging problem due to emerging infectious diseases and the increasing number of multi-drug resistant pathogens. One of the most widespread fungal pathogens is *Aspergillus fumigatus*, responsible for serious and often deadly invasive infections especially in the case of immunocompromised patients. Among antifungals, azoles are the most widely clinically used class. Triazoles, second generation, are today the first-line antifungals in the treatment of aspergillosis.

By combination of *in silico* techniques and methodologies Zoumpoulakis et al. developed new scaffolds endowed with antifungal activity against *A. fumigatus* [25]. After pharmacophore filtering followed by molecular docking, eight compounds were selected and evaluated for antifungal activity. Three of them displayed the highest activity. Their mechanism of action against CYP51 was studied using molecular dynamic simulations. All these compounds exhibited a common binding mode. The results revealed that these three compounds, being different from classical azoles, are the most promising and could be used as lead compounds for further optimization.

## 16. Conditional Oligonucleotide Aptamers as a Powerful Tool for Targeted Cancer Therapy

### *Highlighted by Laura Cerchia* 

Oligonucleotide aptamers, which interact tightly with their targets by recognizing a specific three-dimensional structure, may rival or complement protein antibodies as recognition elements in diagnosis and therapy [26]. The easy and reproducible manufacturing of aptamers combined with their composition that supports a plethora of chemical modifications allowing different conjugation chemistries renders them ideal molecular recognition probes for setting advanced anti-cancer strategies. Recently, Tan’s group, in order to ameliorate the aptamers performance toward an efficacious targeted cancer therapy, developed a strategy to confine the aptamer’s action solely to cancer cells while sparing normal cells, a crucial need when the aptamer’s target is preferentially but not exclusively expressed in tumors [27]. They conjugated the aptamer with PEG5000-azobenzene-NHS, which acts as a caging moiety preventing aptamer-target recognition. When the conjugate reaches the site of the tumor, the hypoxic microenvironment triggers a self-immolative reaction that releases the aptamer competent for binding to its cell surface target. This conditional activation strategy overcoming the potential for the on-target off-tumor effect of aptamers, as well as antibodies, opens a new and powerful avenue for targeted cancer therapy.

## 17. DNA-Based Nanostructures Open the Way to the Discovery of ‘Novel’ Antivirals with Appropriate 3-D Arrangements and Binding Properties

### *Highlighted by Jean-Marc Sabatier* 

In the search for drugs against the Dengue flavivirus, a new approach has been described by Kwon and collaborators [28], which is based on designer DNA nanostructures with some structural characteristics allowing the appropriate spacing and 3-D positioning of the binding sites. They produced a star-shaped DNA scaffold with molecular beacon-like motifs to expose aptamers that strongly recognized the envelope protein domain III clusters located at the viral surface. This compound was a potent antiviral (and binding sensor). Although the design of such bioactive DNA-based multivalent antivirals might be complex, this work opens the way to the development of a new generation of antimicrobial candidates.

## 18. Towards the Synthesis of Highly Effective Treatment of Autoimmune Diseases through Inhibition of Tyrosine Kinase 2

### *Highlighted by Rino Ragno* 

**Figure 3:**
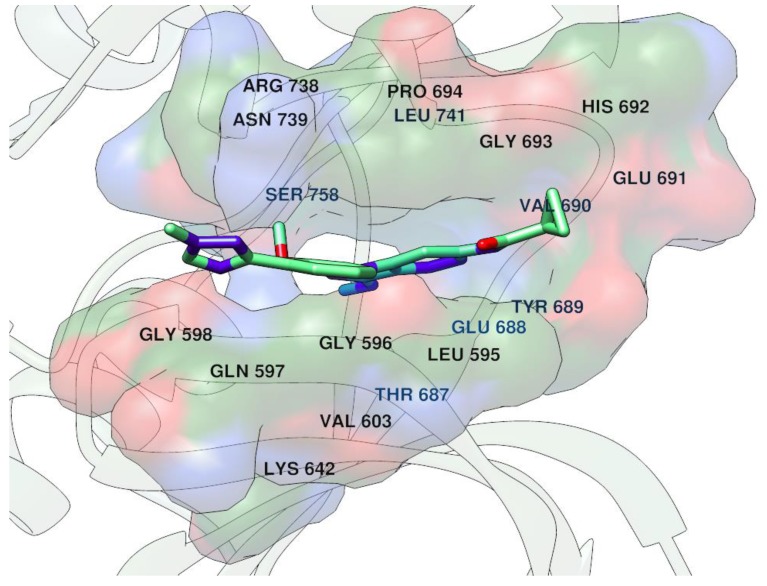
BMS-986165 binding mode into TYK2 JH2 domain (PDB entry code 6NZP).

BMS-986165 is a new allosteric and selective inhibitor of tyrosine kinase 2 (TYK2) recently described by Wrobleski et al. [29]. Different from other Janus family of kinase (JAK) inhibitors, BMS-986165 was demonstrated to be able to allosterically and highly selectively bind the TYK2 pseudokinase or “Janus Homology 2” (JH2) domain. Crystal structure investigation (Figure 3) revealed the unique molecular BMS-986165 binding mode into JH2 domain, revealing the allosteric inhibition. The report is an example of a very important medicinal chemistry result through hit to lead and lead optimization in the very challenging objective to seek for ATP-based kinase selective inhibition across the kinome and especially within the JAK family. BMS-986165 was discovered by means of application of modern approaches and is currently under clinical development as potential treatment for psoriasis, Crohn’s disease and systemic lupus erythematosus. Interestingly, the same group reported a subsequent research article in which BMS-986165 analogues [30] have been deeply investigated as selective allosteric TYK2 inhibitors through in vitro and in vivo experiments, thus giving a strong proof of concept of their achievements.

## 19. A Bayesian Machine Learning Model Developed using Diverse Data Types: A Promising Approach in the Target-Fishing Field

### *Highlighted by Tiziano Tuccinardi* 

Currently, target-fishing approaches are important strategies in medicinal chemistry for identifying the most probable targets of a small molecule. A successful approach would make it possible to clarify the mechanism of action of therapeutically interesting compounds for which the target is still unknown. Furthermore, target-fishing would be useful to predict adverse effects of drug candidates and would also accelerate drug repurposing campaigns. Elemento and co-workers developed BANDIT, a Bayesian ANalysis to determine Drug Interaction Targets, that integrates more than 20,000,000 data points from drug efficacies, post-treatment transcriptional responses, drug structures, reported adverse effects, bioassay results and known targets. As a measurement of the reliability of the model, the authors were able to replicate shared-target relationships, individual drug–target relationships and known mechanisms of action within a predefined test set. Finally, the reliability of the model was experimentally evaluated by confirming new suggested biological activities by using different bioassays and model systems [31]. Given the interesting results obtained, BANDIT represents an important tool that should encourage the development of new computational target-fishing platforms.

## 20. First-in-Class Clinical O-GlcNAcase Inhibitors as a Therapeutic Agent for the Treatment of Tauopathies

### *Highlighted by Andrea Trabocchi* 

**Figure 4:**
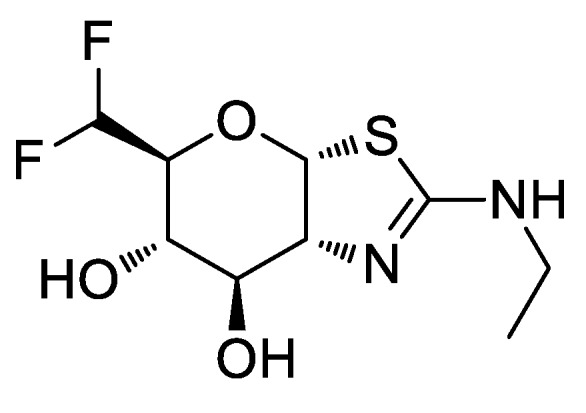
Structure of the O-GlcNAcase inhibitor MK-8719.

Intracellular tau aggregates are a common outcome in a number of neurodegenerative disorders referred to as tauopathies, which are associated with synaptic loss and neuronal death. O-GlcNAcylation in mammalian cells is regulated by the two enzymes glycosyltransferase O-GlcNAc transferase and glycoside hydrolase O-GlcNAcase. O-GlcNAcylation of tau regulates its phosphorylation state, as lower tau phosphorylation is maintained by increased O-GlcNAc modification. Thus, the pharmacological blockade of O-GlcNAcase has been recognized as a therapeutic approach to prevent neurodegeneration brought on by pathological tau by reducing the formation of pathogenic tau aggregates. MK-8719 is a small molecule developed by Merck as an inhibitor of the O-GlcNAcase enzyme, identified through medicinal chemistry and pharmacological studies of a set of molecules based on a previous lead candidate in view of reducing the polar surface area to align the desired drug-like properties [32]. It has been reported that MK-8719 (Figure 4) shows good pharmacokinetic properties with excellent brain penetration, and a robust and sustained pharmacodynamic response, allowing for its selection as a development candidate for first-in-class human phase I clinical trials for the treatment of tauopathies. 

## 21. *2H*-Benzo[*e*][1,2,4]thiadiazin-3(4*H*)-one 1,1-dioxide Scaffold as New Chemotype for Selectively Targeting the Tumor-Associated Carbonic Anhydrases IX and XII 

### *Highlighted by Jean-Yves Winum* 

The membrane bound carbonic anhydrase isoforms hCA IX and hCA XII are considered as therapeutic targets for hypoxic tumors as both of these enzymes are associated with cancer progression, metastasis, and impaired therapeutic response. The development of new and effective inhibitors is driven by the specific targeting of hCA IX/hCA XII over the ubiquitous cytosolic off-target isoforms hCA I and hCA II [33]. Bua and colleagues investigated a new cyclic ureidosulfonamide chemotype based on a 2*H*-benzo[*e*][1,2,4]thiadiazin-3(4*H*)-one 1,1-dioxide scaffold, and resulting from the combination design of the structure of saccharin and acesulfame K [2]. Many derivatives showed enhanced inhibitory activity and selectivity against hCA IX/hCA XII isoforms. In vitro evaluation of anticancer activity revealed an enhanced growth inhibition with a low micromolar IC_50_ against HCT-116 cells under hypoxic conditions. The effect on apoptosis markers in HTC-116 cell lines was an increased level of pro-apoptotic protein expression (Bax, caspase-3 and -9 and pf53) upon treatment with this family of inhibitors [34]. This study paves the way for the drug design and development of new potential candidate drugs targeting tumor-associated carbonic anhydrase isoforms.

## 22. Expanding the Human Proteome with Functionalized Enantiomeric Probes

### *Highlighted by F. Javier Luque* 

Assessing the ‘ligandability’ of proteins is important to explore the ability of the human proteome to bind small molecules, to interrogate the functional role of proteins, and eventually to validate the target druggability in drug discovery. In the search for a methodological approach that enables a global assessment of small-molecule/protein interactions in cells, the authors developed functionalized fragment probes, which include different fragments coupled to photoreactive and bioorthogonal reporter groups, enabling the mapping of >2000 reversible fragment-protein interactions [35]. In this work [36], Parker, Cravatt and coworkers report the next-generation set of functionalized fragment probes, which consist of fragment pairs differing only in absolute stereochemistry. They are intended to provide stronger evidence for the formation of specific stereoselective interactions with proteins in cells. Indeed, using a set of eight pairs of enantiomeric probes, >170 small-molecule/protein interactions were found, including the binding to functionally relevant sites. The disclosure of these stereoselective interactions will be valuable to develop more potent and selective compounds against specific proteins in the cell, leading to more efficient chemical probes and drugs.

## 23. Targeting IL-11 to Halt and Reverse Fibrosis and Inflammation in the Lung

### *Highlighted by Katalin Prokai-Tatrai* 

Patients suffering from incurable idiopathic pulmonary fibrosis (IPF) have abnormal levels of interleukin 11 (IL-11). This protein stimulates fibroblast activation, and therefore, is a critical contributor to fibrosis and inflammation across organs, including the lung. Excessive scarring around the air sacs in the lung eventually leads to respiratory failure and death. Current anti-inflammatory and antifibrotic therapies cannot stop IPF, whose pathophysiology remains to be fully understood. Recently, Ng et al. reported their exciting findings on IL-11 as a therapeutic target for IPF [37]. In vitro mechanistic studies in primary human lung fibroblast cultures were conducted to gain insight into the signaling pathways important for IL-11-driven fibrosis, while in vivo studies used the bleomycin model of pulmonary fibrosis in mice. The research team showed through this model that a neutralizing IL-11 antibody not only diminished lung inflammation but also reversed existing tissue scarring in the animals’ lungs. It is anticipated that IL-11 inhibition using this type of antibody would be devoid of detrimental side effects associated with available antifibrotic therapies, altogether raising hope that the authors’ findings in the mouse model of pulmonary fibrosis can be translated to the curative treatment of IPF patients.

## 24. Unique Tetrapeptides from a *Penicillium* Fungus as Biased Analgesics Targeting the µ-Opioid Receptor

### *Highlighted by Mariana Spetea* 

**Figure 5:**
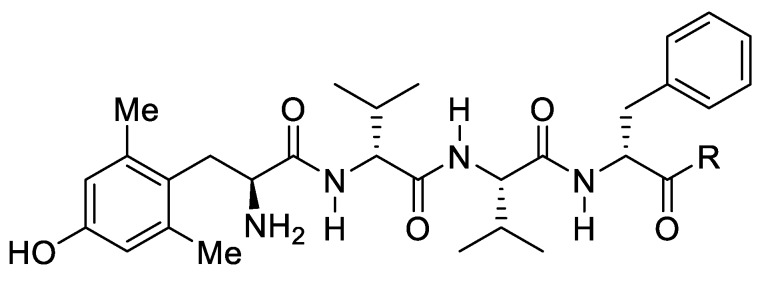
Structures of bilorphin and bilactorphin. Bilorphin, R = NH_2_; Bilactorphin, R = L-Ser(β-Lac)-NH_2_.

The µ-opioid receptor (MOPr) agonists are the most effective analgesics, but their usefulness is limited by serious adverse effects. Medical use and misuse of opioids resulted in a huge rise in opioid-related overdose deaths in the past years. Biased agonism at the MOPr has gained increased importance for opioid drug discovery, where G protein-biased MOPr agonists may deliver analgesia with fewer side effects [38]. Further, compounds derived from natural resources are valuable leads for new drug candidates. Both strategies have been combined by Dekan et al. [39], where discovery of three unusual tetrapeptides with a unique stereochemical arrangement of hydrophobic amino acids from an Australian estuarine isolate of *Penicillium* species is presented. The SAR study led to the design of bilorphin (Figure 5), a potent and selective G protein-biased MOPr agonist. Bilorphin adopts a distinct conformational shape and intermolecular interactions in molecular dynamics simulations. Through the addition of a simple sugar moiety, bilactorphin was generated (Figure 5), as an orally active and effective analgesic in vivo. These findings provide significant knowledge for creating novel pain therapeutics based on microbes, deserving further scientific attention.

## 25. Optimized Peptidic Michael Acceptors for the Treatment of Human African Trypanosomiasis

### *Highlighted by Michael Gütschow* 

Rhodesain, a cathepsin L-like cysteine protease of *Trypanosoma brucei rhodesiense*, represents a promising target for the development of new drugs against the protozoan disease human African trypanosomiasis (HAT), well-known as sleeping sickness. Roberta Ettari at the University of Messina, Italy, and co-workers synthesized a new series of peptidyl vinyl ketones employing a cross-metathesis reaction as key step. The compounds were kinetically characterized to be potent inactivators of rhodesain [40].

**Figure 6:**
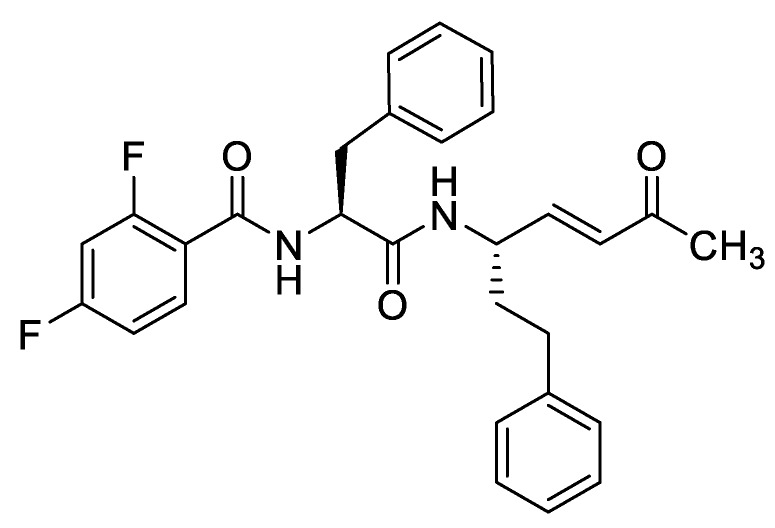
Chemical structure of a new lead for HAT drug discovery.

One of the lead compounds (Figure 6) showed an impressive binding affinity for rhodesain (*K*_i_ = 0.6 pM), a strong inhibitory effectiveness (*k*_2nd_ = *k*_inac_/*K*_i_ = 1,785,000 M^−1^s^−1^), sufficient selectivity towards human cathepsin L, the closest human homolog, as well as good antiparasitic activity as determined using an ATP-monitoring system based on firefly luciferase (EC_50_ = 0.67 μM). Such optimized peptidomimetics certainly represent promising lead compounds for the discovery of new drugs to treat HAT.

## 26. Inhibition of Virulence Factors, Sporulation and Cellular Damage of Auranofin in a Mouse Model Infected with *Clostridioides difficile*

### *Highlighted by Ivan Kosalec* 

As the safety profile, adverse effects and pharmacological activity of medicinal products with marketing authorization is known, repurposing has a bright future, especially for search of new antimicrobials [41]. *Clostridioides difficile* infections (CDI) are increasing in mortality and morbidity in hospital settings, and this opportunistic bacterial pathogen presents a typical outcome of the complex interaction between host-defense and microbial virulence according to the damage-response framework [42]. The work of Hutton et al. [43] demonstrates that auranofin, a drug used orally for rheumatoid arthritis treatment, causes a growth inhibition of vegetative cells, sporulation and toxin production of *C. difficile* in vitro. More importantly, the authors also showed that auranofin could be used as a prevention of CDI in a mouse model, where it led to inhibition of sporulation and toxins A and B production, and importantly to lower epithelial damage, inflammation and edema production, less weight loss and increase in the life span of treated mice. Using the proof-of-concept combining the in vitro and in vivo data, it could be concluded that auranofin is an important candidate for its repurposing for a new indication.

## 27. A New Efficient Positive Allosteric Modulator of α7 Nicotinic Acetylcholine Receptor. New Hope for the Treatment of Patients with Mild to Moderate Alzheimer’s Disease 

### *Highlighed by Catherine Guillou* 

Modulation of α7 nicotinic acetylcholine receptors (nAChRs) by positive allosteric modulators (PAM) is a promising therapeutic approach for the treatment of Alzheimer’s disease (AD), schizophrenia and attention deficit hyperactivity disorder. Researchers from Lupin LTD have identified a novel PAM of α7 nAChR, a thiophenephenylsulfonamide that showed good in vitro potency, with excellent brain penetration and residence time [44]. This compound reverses the cognitive deficits in episodic/working memory in both time-delay and scopolamine-induced amnesia. Additionally, it showed an excellent safety profile in phase 1 and is being evaluated in monotherapy in patients with mild to moderate AD.

## 28. CD9 Is a Marker and a Potential Therapeutic Target for Pancreatic Ductal Adenocarcinoma Tumor-Initiating Cells

### *Highlighed by M. Helena Vasconcelos* 

Pancreatic ductal adenocarcinoma (PDAC) is a very aggressive cancer with increasing incidence; it is difficult to treat and has a poor prognosis. PDAC has high cellular heterogeneity and a high degree of plasticity. The tumor initiating cells or cancer stem cells (CSC) are highly proliferative, have ability to promote metastatic dissemination, and are drug resistant and capable of originating cellular heterogeneity. Nonetheless, so far their identification and targeting has been difficult due to the lack of specific markers. The recent publication by Wang et al. [45] identified the tetraspanin CD9 as a CSC marker for PDAC. Interestingly, CD9 had previously been identified as a CSC marker in glioblastoma [46] and B-cell acute lymphoblastic leukemia [47]. Remarkably, in this study CD9 was also found to enhance glutamine uptake into PDAC cells, allowing altered tumor cell metabolism and supporting PDAC development. Thus, this function of CD9, together with its localization at the cell membrane, makes it an attractive target for therapeutic intervention in PDAC. 

## 29. The FDA-Approved Drug Neratinib Protects Pancreatic Beta Cells in Diabetes

### *Highlighted by George Kokotos* 

**Figure 7:**
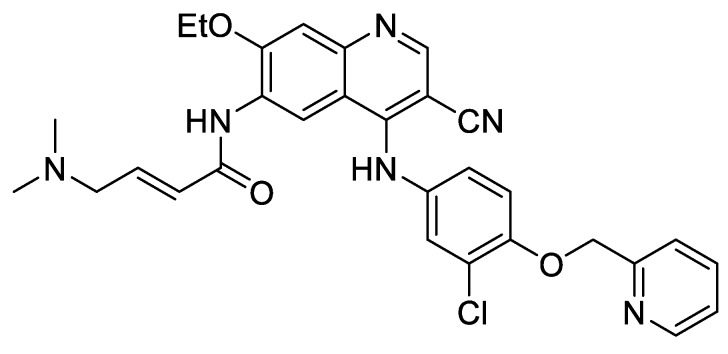
Chemical structure of neratinib.

The discovery of novel agents that may block β-cell apoptosis and at the same time restore β-cell function is of great importance to fight diabetes. Ardestani et al. recently demonstrated that neratinib (Figure 7), an FDA-approved drug targeting HER2/EGFR dual kinases, is a potent mammalian sterile 20-like kinase 1 (MST1) inhibitor, which improves β-cell survival under multiple diabetogenic conditions in human islets and INS-1E cells [48]. Five years ago, it was reported that MST1 is a key regulator of pancreatic β-cell death and dysfunction [49]. The present work shows that neratinib is a previously unrecognized inhibitor of MST1, which is able to attenuate hyperglycemia and improve β-cell function, survival and β-cell mass in type 1 (streptozotocin) and type 2 (obese Leprdb/db) diabetic mouse models. Thus, it represents a potential β-cell-protective drug.

## 30. Human Asparagine Synthetase is a Promising Anticancer Drug Target

### *Highlighted by Giulio Rastelli* 

Asparagine synthetase (ASNS) is an enzyme that catalyzes the ATP-dependent biosynthesis of L-asparagine in cells. Several evidences indicate that ASNS is involved in several types of cancers, but little information is available about this potential drug target. The study by Zhu et al. [50] reports an exemplary approach integrating proteomics, crystallography, computational modelling and molecular biology to clarify important mechanistic and structural information about this target. First, the authors demonstrated that one selected methylsulfoximine inhibitor exhibits excellent selectivity in HCT-116 cell lysates, with almost no off-target binding. Then, they determined the first high-resolution crystal structure of human ASNS, which was used to perform docking, molecular dynamics and free-energy perturbation studies of enzyme–ligand complexes. The resulting binding modes were used to select site-directed point mutations to map and probe the ligand binding site. Overall, this study sets the basis for the design and discovery of a second generation of potent, selective and more drug-like inhibitors of human ASNS to be developed as drugs against metastasis.

## 31. Visible Light Photoactivatable Prodrugs: Design of the Platinum Magic Bullet 

### *Highlighted by Maria Emília de Sousa* 

Photoactivatable molecules are considered in strategic advances in medicinal chemistry [51]. The concept of light liberating a photolabile protecting group has been used in photodynamic therapy or in prodrugs to control the activity of a bioactive compound in a more precise temporal release for specific effects. Zhigang Wang et al. [52] reported the design of chemotherapeutic “magic bullets” that can be controllably activated by red light, allowing high penetration. The group developed phorbiplatin a stable mutual prodrug of oxaliplatin, a first-line clinical antineoplastic, and pyropheophorbide (PPA), a photo-absorber that, acting as carrier, has also the function of photosensitizer. A unique photoreduction mechanism is proposed to quickly and efficiently activate oxaliplatin in a spatial and temporal fashion. Phorbiplatin is able to kill tumor cells much more efficiently than the mixture of the parent drug with PPA in a mouse tumor model.

**Figure 8:**
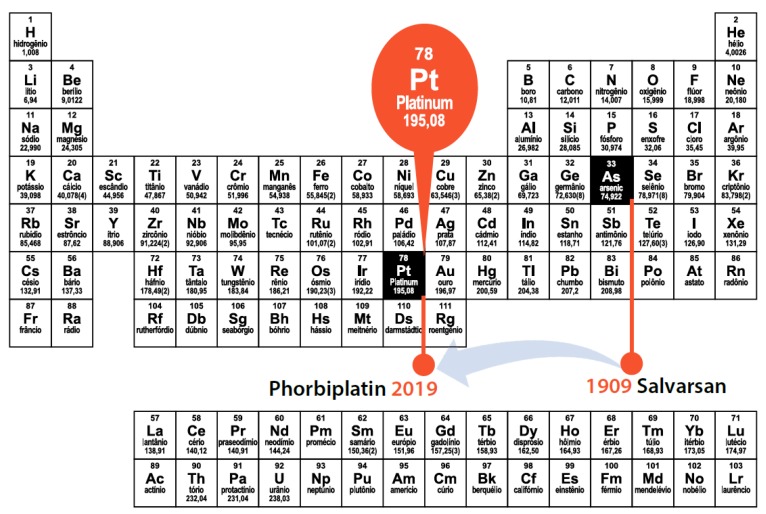
A spatial and temporal breakthrough with the discovery of phorbiplatin.

A spatial and temporal breakthrough was made due to the first arsenic-based magic bullet to bring light into the commemorations of the 150th anniversary of the Periodic Table (Figure 8).

## 32. Allosteric Modulators of Cannabinoid Receptor 1: Developing Compounds for Improved Functional Diversity

### *Highlighted by Clementina Manera* 

CB1 cannabinoid receptor (CB1R) is well-known as a target for the treatment of several pathologies. However, its direct modulation by agonists or antagonists/inverse agonists has been associated with undesirable effects on the central nervous system (CNS), thus limiting the clinical development of such agents. Allosteric modulators (AMs) represent a promising approach to achieving the potential therapeutic benefits of the orthosteric CB1R ligands, while avoiding their side-effects. In particular, agonist-positive allosteric modulators (ago-PAMs) that increase potency and/or efficacy of orthosteric agonists, as well as displaying intrinsic efficacy, may be suitable for treating diseases whose etiology involves severe loss of endogenous neurotransmitters.

Recently, the application of fluorine- and nitrogen-walk approaches to the 2-phenylindole scaffold permitted the identification of two key compounds with high ago-PAM activity, good lipophilicity and drug-like physicochemical properties [53]. Both compounds also exhibited good in vivo potency in a preclinical inflammatory pain model with long-lasting action without adverse cannabimimetic effects and therefore they could be worth investigating in select CNS disorders. Finally, the observed SAR trend was accounted for by using a recently developed computational model for the CB1R allosteric agonism [54]. 

## 33. Inhibition of Trypanothione Reductase by 4,15-*iso*-Atriplicolide Tiglate: Mode of Inhibition and Structural Requirements

### *Highlighted by Sandra Gemma* 

Natural products are an invaluable source of new drugs and lead compounds against a plethora of diseases, with enormous contribution to the discovery of antibacterial and antiparasitic drugs. Sesquiterpene lactones (STLs) have been demonstrated to have potent and, in many instances, selective toxicity against protozoan parasites, the etiological agents of several neglected tropical diseases (NTDs). Recently, furanoheliangolide-type STLs, characterized by the presence of Michael-acceptor structural motifs, were demonstrated to be endowed with high-level in vitro activity against *Trypanosoma brucei rhodesiense* [55]. In their work, Lenz and colleagues [56] studied the molecular basis of the activity of different furanoheliangolides. The STL 4,15-*iso*-atriplicolide tiglate and its homologues were demonstrated to inhibit trypanothione reductase (TR), an enzyme playing a critical role in the metabolism and survival of trypanosomatids. The authors also highlighted the structural requirements necessary for the irreversible TR inactivation by this class of STLs. This study adds a key piece of information toward the full therapeutic exploitation of STLs in the field of human African trypanosomiasis and related NTDs.

## 34. The *Pseudomonas aeruginosa* Importer PfeA-Fe^3+^-Enterobactin Complex Indicates New Ways to Overcome Bacterial Resistance to Antibiotics

### *Highlighted by Stefano Mangani* 

Iron is an essential nutrient for bacteria that have evolved siderophore molecules to chelate Fe^3+^ ions from insoluble ferric compounds present in the environment. In Gram-negative bacteria Fe^3+^-siderophore complexes are recognized by selective outer-membrane TonB-dependent transporters [57] that bind the complex and translocate it into the periplasmic space. One of the mechanisms of bacterial resistance to antibiotics are the multidrug efflux pumps [58]. To overcome this aspect of bacterial resistance, a “Trojan horse” strategy has been conceived, consisting of attaching an antibiotic molecule to a siderophore [59]. An international research team has recently established the essential role played by an extracellular recognition site of the PfeA importer of the pathogen *Pseudomonas aeruginosa* in binding the Fe^3+^-enterobactin (a secreted siderophore) complex [60]. The binding of the Fe^3+^-enterobactin complex to this site is the obligatory event leading to the import of the siderophore into the bacterium periplasmic space. This study indicates that specifically designed siderophore-antibiotic complexes, targeting the enterobactin-dependent iron-uptake system of *P. aeruginosa* constitutes a promising approach to fight antibiotic resistance by this threatening pathogen.

## 35. Anticancer Phosphonodiesters Containing Nature Inspired Molecular Scaffolds

### *Highlighted by Carlo Siciliano* 

Great efforts are dedicated to the synthesis of new chemotherapeutic drugs with optimal characteristics, and molecular structures inspired by natural scaffolds, e.g. phenyl moieties, as found in known powerful antioxidants. A recent work [61] reports on the design of a series of interesting anticancer candidates, containing a 2,6-diaminopyridine scaffold functionalized with a phosphonate ester moiety linked to sterically hindered dialkylated phenol rings. The synthesis is very simple and based on the heteroaromatic electrophilic substitution of the 2,6-diaminopyridine lead core with aryl 3,5-di-*tert*-butyl-4-oxocyclohexa-2,5-dienylidene methylphosphonates. All compounds were extensively tested for their biological activities. Antioxidant, antimicrobial (bacteriostatic and fungistatic), cytotoxicity, and haemolytic tests were completed by a multiplex analysis on apoptosis markers, establishing the role of the internal pathway of caspase-9 activation. The natural dialkylated oxyphenol ring is mandatory for the biological activity. None of the compounds showed antimicrobial or haemolytic activity, but in vitro tests, using normal Chang liver cells as the reference, highlighted the high efficiency of all compounds against a variety of tumor cell cultures (breast adenocarcinoma, epithelioid and human lung carcinoma, human prostate cancer), with a totally negative cytotoxicity.

## 36. A Bioinspired Technology as an Antitumor Drug Carrier: Enzymatic Self-Assemblies

### *Highlighted by Stefania Galdiero* 

The development of amphiphilic peptides, in combination with an enzyme-responsive self-assembly are of great interest in biomedicine and biotechnology and could lead to nanomaterials having great potential as vectors for antitumor therapeutics. Gong et al. designed a positively charged amphiphilic peptide, A_6_K_2_, able to stably self-assemble in nanovesicles, which slowly break down and gradually form nanofibres following enzyme catalysis [62]. Furthermore, the lipid-soluble antitumor drug doxorubicin (DOX) could be loaded into the hydrophobic core of the nanovesicles yielding nanomaterials with outstanding antibacterial and antitumor properties, low cytotoxicity to normal mammalian cells and excellent selectivity for cancer cells. They developed an innovative and fascinating approach to attack tumor cells and cause tumor cell death, which suggests that nanomaterials with great potential in drug delivery for biomedical applications are on their way.

## 37. MOST: A New Strategy with High Precision to Pry into the Vasculature-Dependent Pathogenesis of Alzheimer’s Disease

### *Highlighted by Hong Liu* 

Mounting evidence reveals that Alzheimer’s disease (AD) progression and severity are highly associated with the structural and functional integrity of the cerebral vasculature. Efforts have been made to obtain the 3D atlases of the vasculature for whole-brain samples on a mesoscopic level. However, high resolution and large sample size are mutually exclusive in conventional imaging methods. Recently, by using micro-optical sectioning tomography (MOST), a study has successfully generated cross-scale whole-brain 3D atlases that cover the entire vascular system from large vessels down to smallest capillaries at submicron resolution [63]. Meanwhile, its characterization of the hippocampus has demonstrated that it is more susceptible to vasculature dysfunction due to a lower mean vascular diameter, volume fraction and length density. The study also compared the vasculature morphology of both wild-type mice and APPsew/PSEN1dE9 transgenic (Tg-AD) mice and discovered that the reduction of the mean vessel diameter, vascular-volume fraction and average branch angle leads to poor blood perfusion in the hippocampus of Tg-AD mice. Overall, this study provides a new insight on vasculature-related AD pathogenesis and a potential therapeutic approach for AD.

## 38. Accessing New PET Radiotracers with Copper-Mediated Radiofluorination: Radiosynthesis of [^18^F]Atorvastatin

### *Highlighted by Peter J. H. Scott* 

**Figure 9:**
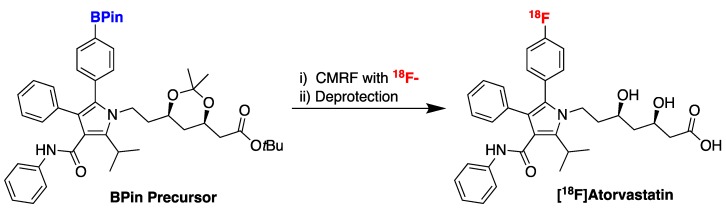
Radiosynthesis of [^18^F]atorvastatin.

Positron emission tomography (PET), a functional imaging technique using bioactive molecules tagged with PET radionuclides such as ^18^F (radiopharmaceuticals), is increasingly used by pharmaceutical companies to accelerate drug discovery [64]. PET can provide quantitative information on target engagement (which can inform dose projection), as well as any off-target binding that might present safety, toxicity and/or side effect concerns. Historically, use of PET in such applications has been limited because of difficulties in labeling pharmaceutical assets with ^18^F. For example, cholesterol-lowering statins are amongst the biggest-selling drugs worldwide but there are limited examples of ^18^F-labeled versions being used to support their development. Recently however, copper-mediated radiofluorination (CMRF) has greatly expanded the chemical space accessible for labeling with ^18^F [65]. Reflecting on this, Elsinga recently employed CMRF to synthesize [^18^F]atorvastatin from a pinacol boronate ester (BPin) precursor (Figure 9) [66]. As one of the most complex substrates labeled with CMRF to date, the work highlights important potentialities and limitations of CMRF going forward. The team obtained [^18^F]atorvastatin in ~2% radiochemical yield, making this important radiopharmaceutical available for researchers in the cholesterol treatment space.

## 39. Fighting Hypoxic Tumors with Photoactivated Organometallics

### *Highlighted by Cristóbal de los Ríos* 

**Figure 10:**
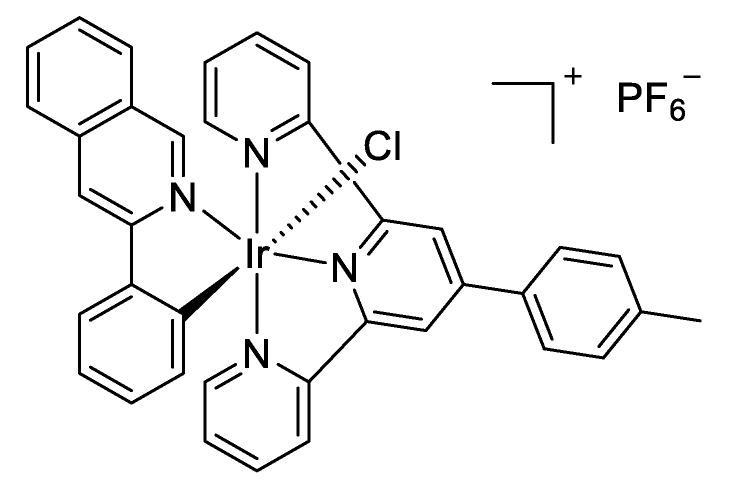
Chemical structure of the Ir complex evaluated by Sadler and co-workers.

Photodynamic therapy with metal complexes has recently emerged as an interesting approach to avoid chemoresistance and side effects in cancer. However, current photoactivated metal complexes need O_2_ to exert their action. Thus, they lose activity in cancer cells growing in hypoxic environments, which is a rather common situation. To solve this problem, an interesting paper has reported the chemotherapeutic profile of an iridium complex effective in both hypoxia and normoxia [67]. The key step is the photocatalytical oxidation of NADH, which is essential for cell proliferation under hypoxia, so its disruption leads to cancer cell death. This iridium complex (Figure 10) was fully characterized by X-ray, as well as by its electro and photochemical properties. It presents phosphorescence when irradiated at 463 nm, and is stable under dark or light conditions. By DFT, the authors hypothesize a π-π adduct between NADH and the excited-state Ir complex, facilitating NADH oxidation by one-electron transfer. Under light irradiation, it noticeably reduced cell viability of several cancer cell lines in both hypoxia and normoxia. Cisplatin was much less potent in similar cell lines under hypoxia.

## 40. Enzymatic Preparation of 2’3’-Cyclic Dinucleotides and Their Binding to STING Adaptor Protein

### *Highlighted by Luigi A. Agrofoglio* 

The cyclic dinucleotide-cGAS–STING axis plays important roles in host immunity, DNA, and RNA virus-sensing. Cyclic dinucleotides (CDNs) are natural substrates that directly activate STING signaling. Indeed, numerous synthetic routes for 2’3’- and 3’3’CDNs have been explored but are suffering from complex and multistep syntheses. Researchers from Czech Republic have investigated the use of dinucleotide synthases for 2’3’CDN preparation [68]. CDNs are anchored to STING protein through π–π and cation–π interactions. They have identified the most suitable enzyme for this purpose and obtained 33 CDNs (modified at the heterocycle moiety), and determined SAR between STING and CDNs by computational chemistry and QM/MM calculations. These data have great potential to design small molecules as well as new CDNs able to activate (or not) the cGAS-STING pathway.

## 41. Pteridine-Reductase-1 Inhibitors as a New Weapon against *Trypanosoma brucei* Infections

### *Highlighted by Simona Collina* 

*Trypanosoma brucei is* the causative agent of the neglected human African trypanosomiasis (HAT) disease. Current drug treatments suffer from host toxicity and emerging resistance. An attractive start point for discovering new drugs against this neglected disease is targeting enzymes essential for the parasite. The finding that the pteridine reductase enzyme is essential for parasite survival offers an opportunity to exploit a known, druggable target for the treatment of HAT.

Landi et al. [69] exploited cycloguanil as a scaffold for the development of novel pteridine-reductase-1 (PTR1) inhibitors. The elucidation of the binding mode supported the rational design of novel 2,4-diamino-1,6-dihydrotriazine derivatives, thus identifying two new potent PTR1 inhibitors as a valuable starting point for the development of dual PTR1 and dihydrofolate reductase (DHFR) inhibitors with antiparasitic activity. The simultaneous inhibition of DHFR and PTR1 in *T. brucei* is a promising new strategy for the treatment of HAT and the novel 1,6-dihydrotriazines represent new molecular tools to develop potent dual PTR1 and DHFR inhibitors. Moreover, the similarity of the target structures among the different organisms suggests that the triazine core can be developed further for pan-PTR1-enzyme inhibition.

## 42. Exploring and Targeting Protein–Protein Druggable Cavities

### *Highlighted by Rita C. Guedes* 

There are more than 645,000 reported disease-relevant protein–protein interactions (PPIs) in the human interactome. However, historically PPIs were regarded as “undruggable” targets, an idea that was challenged by recent clinical successes and opened a totally new era in drug discovery research. Today, several PPI modulators are under clinical trials for a broad range of therapeutic areas, with a special focus in oncology. PPIs are difficult to modulate, especially with small molecules, since they involve large and flat protein surfaces lacking the deep and well-defined binding pockets, suitable to bind a ligand with high affinity, found in the “traditional” targets of most currently existing therapeutic drugs.

Rognan and coworkers [70] developed a fully automated structure-based workflow, to identify and prioritize druggable cavities. In this work, the entire Protein Data Bank was exploited to identify PPI interfaces and different types of druggable cavities in protein–ligand complexes, and based on their properties and spatial locations, classify them (interfacial, rim, allosteric, orthosteric). A systematic comparison between the pocket cavities identified from the Protein Data Bank protein-complexes and PPI cavities was performed (http://drugdesign.unistra.fr/ppiome).

## 43. The Enemy of My Enemy Is My Friend: A New Antibiotic Selective for Gram-Negative Pathogens Isolated from Nematode Microbiome Symbionts

### *Highlighted by Diego Muñoz-Torrero* 

**Figure 11:**
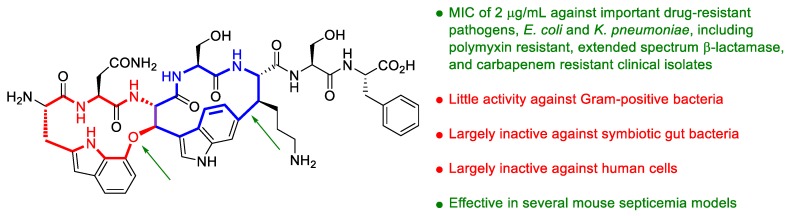
Chemical structure of darobactin, featuring two fused rings (in red and blue) with an unprecedented aromatic-aliphatic ether linkage between two tryptophans and a unique tryptophan-lysine bond between two inactivated carbons (green arrows).

Following the logic that antimicrobial compounds could be found in bacteria that share similar requirements for antibiotics with humans, the group of Kim Lewis has discovered darobactin, the first member of a new class of antibiotics that selectively kills Gram-negative bacteria (GNB) [71]. GNB that are common opportunistic pathogens of humans are abundant in the gut microbiome of entomopathogenic nematodes, where GNB are competitors of nematophilic symbiotic bacteria. It was reasoned that nematode symbionts could release antimicrobials to defend themselves against GNB. Upon a screening of a library of nematode symbionts (*Photorhabdus* and *Xenorhabdus* strains) against *Escherichia coli*, they identified darobactin, a modified heptapeptide with an unusual structure (Figure 11) and an unusual mode of action on an essential outer membrane protein (BamA). Darobactin is effective against important GNB in vitro and in several mouse septicemia models, with little or no activity on Gram-positive bacteria, gut commensals, and human cell lines. A good opportunity to replenish the reduced pipeline of antibiotics against drug-resistant GNB!

## References

[1] Loupias P., Dechamps-Olivier I., Dupont L., Vanlemmens P., Mullié C., Taudon N., Bouchut A., Dassonville-Klimpt A., Sonnet P. (2019). Study of iron piperazine-based chelators as potential siderophore mimetic. Pharmaceuticals.

[2] Cummings J., Lee G., Ritter A., Sabbagh M., Zhong K. (2019). Alzheimer’s disease drug development pipeline: 2019. Alzheimers Dement..

[3] Scheiner M., Dolles D., Gunesch S., Hoffmann M., Nabissi M., Marinelli O., Naldi M., Bartolini M., Petralla S., Poeta E. (2019). Dual-acting cholinesterase-human cannabinoid receptor 2 ligands show pronounced neuroprotection in vitro and overadditive and disease-modifying neuroprotective effects In Vivo. J. Med. Chem..

[4] Watkins P.B., Zimmerman H.J., Knapp M.J., Gracon S.I., Lewis K.W. (1994). Hepatotoxic effects of tacrine administration in patients with Alzheimer’s disease. JAMA.

[5] Dash R.P., Tichý T., Veeravalli V., Lam J., Alt J., Wu Y., Tenora L., Majer P., Slusher B.S., Rais R. (2019). Enhanced oral bioavailability of 2-(phosphonomethyl)-pentanedioic acid (2-PMPA) from its (5-methyl-2-oxo-1,3-dioxol-4-yl)methyl (ODOL)-based prodrugs. Mol. Pharm..

[6] Blache P., Canterel-Thouennon L., Busson M., Verdié P., Subra G., Ychou M., Prévostel C. (2019). A short SOX9 peptide mimics SOX9 tumor suppressor activity and is sufficient to inhibit colon cancer cell growth. Mol. Cancer Ther..

[7] Feagan B.G., Sandborn W.J., Gasink C., Jacobstein D., Lang Y., Friedman J.R., Blank M.A., Johanns J., Gao L.L., Miao Y. (2016). Ustekinumab as induction and maintenance therapy for Crohn’s disease. N. Engl. J. Med..

[8] Li K., Friedman J.R., Chan D., Pollack P., Yang F., Jacobstein D., Brodmerkel C., Gasink C., Feagan B.G., Sandborn W.J. (2019). Effects of ustekinumab on histologic disease activity in patients with Crohn’s disease. Gastroenterology.

[9] Whitehouse A.J., Libardo M.D.J., Kasbekar M., Brear P.D., Fischer G., Thomas C.J., Barry C.E., Boshoff H.I.M., Coyne A.G., Abell C. (2019). Targeting of fumarate hydratase from *Mycobacterium tuberculosis* using allosteric inhibitors with a dimeric-binding mode. J. Med. Chem..

[10] Yang Z., Wang D., Johnsoni J.K., Pascal L.E., Takubo K., Avula R., Chakka A.B., Zhou J., Chen W., Zhong M. (2019). A novel small molecule targets androgen receptor and its splice variants in castration-resistant prostate cancer. Mol. Cancer Ther..

[11] Rheumatoid Arthritis, National Institutes of Health. https://www.niams.nih.gov/health-topics/rheumatoid-arthritis.

[12] Micheli L., Bozdag M., Akgul O., Carta F., Guccione C., Bergonzi M.C., Bilia A.R., Cinci L., Lucarini E., Parisio C. (2019). Pain relieving effect of-NSAIDs-CAIs hybrid molecules: Systemic and intra-articular treatments against rheumatoid arthritis. Int. J. Mol. Sci..

[13] Ji M.J., Hong J.H. (2019). An overview of carbonic anhydrases and membrane channels of synoviocytes in inflamed joints. J. Enzyme Inhib. Med. Chem..

[14] Bua S., Lucarini L., Micheli L., Menicatti M., Bartolucci G., Selleri S., Di Cesare Mannelli L., Ghelardini C., Masini E., Carta F. (2019). Bioisosteric development of multitarget nonsteroidal anti-inflammatory drug-carbonic anhydrases inhibitor hybrids for the management of rheumatoid arthritis. J. Med. Chem..

[15] Wand X., Sun G., Feng T., Zhang J., Huang X., Wang T., Xie Z., Chu X., Yang J., Wang H. (2019). Sodium oligomannate therapeutically remodels gut microbiota and suppresses gut bacterial amino acids-shaped neuroinflammation to inhibit Alzheimer’s disease progression. Cell Res..

[16] Lin A., Giuliano C.J., Palladino A., John K.M., Abramowicz C., Yuan M.L., Sausville E.L., Lukow D.A., Liu L., Chait A.R. (2019). Off-target toxicity is a common mechanism of action of cancer drugs undergoing clinical trials. Sci. Transl. Med..

[17] Lipinski C.A., Lombardo F., Dominy B.W., Feeney P.J. (1997). Experimental and computational approaches to estimate solubility and permeability in drug discovery and development settings. Adv. Drug Deliv. Rev..

[18] Mignani S., Rodrigues J., Tomas H., Jalal R., Singh P.P., Majoral J.P., Vishwakarma R.A. (2018). Present drug-likeness filters in medicinal chemistry during the hit and lead optimization process: How far can they be simplified?. Drug Discov. Today.

[19] Gupta M., Lee H.J., Barden C.J., Weaver D.F. (2019). The blood-brain barrier (BBB) score. J. Med. Chem..

[20] Fulp J., He L., Toldo S., Jiang Y., Boice A., Guo C., Li X., Rolfe A., Sun D., Abbate A. (2018). Structural insights of benzenesulfonamide analogues as Nlrp3 inflammasome inhibitors: Design, synthesis, and biological characterization. J. Med. Chem..

[21] Jiang Y., He L., Green J., Blevins H., Guo C., Harsiddhbhai Patel S., Halquist M.S., McRae M.P., Venitz J., Wang X.Y. (2019). Discovery of second-generation NLRP3 inflammasome inhibitors: Design, synthesis, and biological characterization. J. Med. Chem..

[22] De Cunto G., Brancaleone V., Riemma M.A., Cerqua I., Vellecco V., Spaziano G., Cavarra E., Bartalesi B., D’Agostino B., Lungarella G. (2019). Functional contribution of sphingosine-1-phosphate to airway pathology in cigarette smoke-exposed mice. Br. J. Pharmacol..

[23] Rahman I., De Cunto G., Sundar I.K., Lungarella G. (2017). Vulnerability and genetic susceptibility to cigarette smoke-induced emphysema in mice. Am. J. Respir. Cell Mol. Biol..

[24] Tashkin D.P., Altose M.D., Connett J.E., Kanner R.E., Lee W.W., Wise R.A. (1996). Methacholine reactivity predicts changes in lung function over time in smokers with early chronic obstructive pulmonary disease. The Lung Health Study Research Group. Am. J. Respir. Crit. Care Med..

[25] Kritsi E., Matsoukas M.-T., Potamitis C., Detsi A., Ivanov M., Sokovic M., Zoumpoulakis P. (2019). Novel hit compounds as putative antifungals: The case of *Aspergillus fumigatus*. Molecules.

[26] Zhou J., Rossi J. (2017). Aptamers as targeted therapeutics current potential and challenges. Nat. Rev. Drug Discov..

[27] Zhou F., Fu T., Huang Q., Kuai H., Mo L., Liu H., Wang Q., Peng Y., Han D., Zhao Z. (2019). Hypoxia-activated PEGylated conditional aptamer/antibody for cancer imaging with improved specificity. J. Am. Chem. Soc..

[28] Kwon P.S., Ren S., Kwon S.J., Kizer M.E., Kuo L., Xie M., Zhu D., Zhou F., Zhang F., Kim D. (2020). Designer DNA architecture offers precise and multivalent spatial pattern-recognition for viral sensing and inhibition. Nat. Chem..

[29] Wrobleski S.T., Moslin R., Lin S., Zhang Y., Spergel S., Kempson J., Pitts W.J., Tokarski J., Strnad J., Shuster D. (2019). Highly selective inhibition of tyrosine kinase 2 (TYK2) for the treatment of autoimmune diseases: Discovery of the allosteric inhibitor BMS-986165. J. Med. Chem..

[30] Moslin R., Zhang Y., Wrobleski S.T., Lin S., Mertzman M., Tokarski J.S., Strnad J., Gillooly K., McIntyre K.W., Zupa-Fernandez A. (2019). Identification of *N*-methyl nicotinamide and *N*-methyl pyridazine-3-carboxamide pseudokinase domain ligands as highly selective inhibitors of tyrosine kinase 2 (TYK2). J. Med. Chem..

[31] Madhukar N.S., Khade P.K., Huang L., Gayvert K., Galletti G., Stogniew M., Allen J.E., Giannakakou P., Elemento O. (2019). A Bayesian machine learning approach for drug target identification using diverse data types. Nat. Commun..

[32] Selnick H.G., Hess J.F., Tang C., Liu K., Schachter J.B., Ballard J.E., Marcus J., Klein D.J., Wang X., Pearson M. (2019). Discovery of MK-8719, a potent O-GlcNAcase inhibitor as a potential treatment for tauopathies. J. Med. Chem..

[33] Supuran C.T., Alterio V., Di Fiore A., D’ Ambrosio K., Carta F., Monti S.M., De Simone G. (2018). Inhibition of carbonic anhydrase IX targets primary tumors, metastases, and cancer stem cells: Three for the price of one. Med. Res. Rev..

[34] Bua S., Lomelino C.L., Murray A.B., Osman S.M., Alothman Z.A., Bozdag M., Aziz H.A.A., Eldehna W.M., McKenna R., Nocentini A. (2019). “A sweet combination”: Developing saccharin and acesulfame K structures for selectively targeting the tumor-associated carbonic anhydrases IX and XII. J. Med. Chem..

[35] Parker C.G., Galmozzi A., Wang Y., Correia B.E., Sasaki K., Joslyn C.M., Kim A.S., Cavallaro C.L., Lawrence R.M., Johnson S.R. (2017). Ligand and target discovery by fragment-based screening in human cells. Cell.

[36] Wang Y., Dix M.M., Biando G., Remsberg J.R., Lee H.-Y., Kalocsay M., Gygi S.P., Forli S., Vite G., Lawrence R.M. (2019). Expedited mapping of the ligandable proteome using fully functionalized enantiomeric probe pairs. Nat. Chem..

[37] Ng B., Dong J., D’Agostino G., Viswanathan S., Widjaja A.A., Lim W.-W., Ko N.S.J., Tan J., Chothani S.P., Huang B. (2019). Interleukin-11 is a therapeutic target in idiopathic pulmonary fibrosis. Sci. Trans. Med..

[38] Turnaturi R., Chiechio S., Salerno L., Rescifina A., Pittalà V., Cantarella G., Tomarchio E., Parenti C., Pasquinucci L. (2019). Progress in the development of more effective and safer analgesics for pain management. Eur. J. Med. Chem..

[39] Dekan Z., Sianati S., Yousuf A., Sutcliffe K.J., Gillis A., Mallet C., Singh P., Jin A.H., Wang A.M., Mohammadi S.A. (2019). A tetrapeptide class of biased analgesics from an Australian fungus targets the µ-opioid receptor. Proc. Natl. Acad. Sci. USA.

[40] Ettari R., Previti S., Maiorana S., Amendola G., Wagner A., Cosconati S., Schirmeister T., Hellmich U.A., Zappalà M. (2019). Optimization strategy of novel peptide-based Michael acceptors for the treatment of human African trypanosomiasis. J. Med. Chem..

[41] Pushpakom S., Iorio F., Eyers P.A., Escott K.J., Hopper S., Wells A., Doig A., Guilliams T., Latimer J., McNamee C. (2019). Drug repurposing: Progress, challenges and recommendations. Nat. Rev. Drug Discov..

[42] Casadevall A., Pirofski L.A. (2003). The damage-response framework of microbial pathogenesis. Nat. Microbiol. Rev..

[43] Hutton M.L., Pehlivanoglu H., Vidor C.J., James M.L., Thomson M.J., Lyras D. (2019). Repurposing auranofin as a *Clostridioides difficile* therapeutic. J. Antimicrob. Chemother..

[44] Sinha N., Karche N.P., Verma M.K., Walunj S.S., Nigade P.B., Jana G., Kurhade S.P., Hajare A.K., Tilekar A.R., Jadhav G.R. (2019). Discovery of novel, potent, brain-permeable, and orally efficacious positive allosteric modulator of α7 nicotinic acetylcholine receptor [4-(5-(4-chlorophenyl)-4-methyl-2-propionylthiophen-3-yl)benzenesulfonamide]: Structure−activity relationship and preclinical characterization. J. Med. Chem..

[45] Wang V.M.-Y., Ferreira R.M.M., Almagro J., Evan T., Legrave N., Zaw Thin M., Frith D., Carvalho J., Barry D.J., Snijders A.P. (2019). CD9 identifies pancreatic cancer stem cells and modulates glutamine metabolism to fuel tumour growth. Nat. Cell Biol..

[46] Podergajs N., Motaln H., Rajčević U., Verbovšek U., Koršič M., Obad N., Espedal H., Vittori M., Herold-Mende C., Miletic H. (2016). Transmembrane protein CD9 is glioblastoma biomarker, relevant for maintenance of glioblastoma stem cells. Oncotarget.

[47] Yamazaki H., Xu C.W., Naito M., Nishida H., Okamoto T., Ghani F.I., Iwata S., Inukai T., Sugita K., Morimoto C. (2011). Regulation of cancer stem cell properties by CD9 in human B-acute lymphoblastic leukemia. Biochem. Biophys. Res. Commun..

[48] Ardestani A., Li S., Annamalai K., Lupse B., Geravandi S., Dobrowolski A., Yu A., Zhu S., Baguley T.D., Surakattula M. (2019). Neratinib protects pancreatic beta cells in diabetes. Nat. Commun..

[49] Ardestani A., Paroni F., Azizi Z., Kaur S., Khobragade V., Yuan T., Frogne T., Tao W., Oberholzer J., Pattou F. (2014). MST1 is a key regulator of beta cell apoptosis and dysfunction in diabetes. Nat. Med..

[50] Zhu W., Radadiya A., Bisson C., Wenzel S., Nordin B.E., Martínez-Márquez F., Imasaki T., Sedelnikova S.E., Coricello A., Baumann P. (2019). High-resolution crystal structure of human asparagine synthetase enables analysis of inhibitor binding and selectivity. Commun. Biol..

[51] Wu G., Zhao T., Kang D., Zhang J., Song Y., Namasivayam V., Kongsted J., Pannecouque C., De Clercq E., Poongavanam V. (2019). Overview of recent strategic advances in medicinal chemistry. J. Med. Chem..

[52] Wang Z., Wang N., Cheng S.-C., Xu K., Deng Z., Chen S., Xu Z., Xie K., Tse M.-K., Shi P. (2019). Phorbiplatin, a highly potent Pt(IV) antitumor prodrug that can be controllably activated by red light. Chem.

[53] Garai S., Kulkarni P.M., Schaffer P.C., Leo L., Brandt A.L., Zagzoog A., Black T., Lin X., Hurst D.P., Janero D.R. (2019). Application of fluorine- and nitrogen-walk approaches: Defining the structural and functional diversity of 2-phenylindole class of CB1 receptor positive allosteric modulators. J. Med. Chem..

[54] Hurst D.P., Garai S., Kulkarni P.M., Schaffer P.C., Reggio P.H., Thakur G.A. (2019). Identification of CB1 receptor allosteric sites using force-biased MMC simulated annealing and validation by structure-activity relationship studies. ACS Med. Chem. Lett..

[55] Schmidt T.J., Da Costa F.B., Lopes N.P., Kaiser M., Brun R. (2014). In silico prediction and experimental evaluation of furanoheliangolide sesquiterpene lactones as potent agents against *Trypanosoma brucei rhodesiense*. Antimicrob. Agents Chemother..

[56] Lenz M., Krauth-Siegel R.L., Schmidt T.J. (2019). Natural sesquiterpene lactones of the 4,15-iso-atriplicolide type are inhibitors of trypanothione reductase. Molecules.

[57] Noinaj N., Guillier M., Barnard T.J., Buchanan S.K. (2010). TonB-dependent transporters: Regulation, structure, and function. Annu. Rev. Microbiol..

[58] Alcalde-Rico M., Hernando-Amado S., Blanco P., Martínez J.L. (2016). Multidrug efflux pumps at the crossroad between antibiotic resistance and bacterial virulence. Front. Microbiol..

[59] Schalk I.J., Mislin G.L.A. (2017). Bacterial iron uptake pathways: Gates for the import of bactericide compounds. J. Med. Chem..

[60] Moynié L., Milenkovic S., Mislin G.L.A., Gasser V., Malloci G., Baco E., McCaughan R.P., Page M.G.P., Schalk I.J., Ceccarelli M. (2019). The complex of ferric-enterobactin with its transporter from *Pseudomonas aeruginosa* suggests a two-site model. Nat. Commun..

[61] Gibadullina E., Nguyen T.T., Strelnik A., Sapunova A., Voloshina A., Sudakov I., Vyshtakalyuk A., Voronina J., Pudovik M., Burilov A. (2019). New 2,6-diaminopyridines containing a sterically hindered benzylphosphonate moiety in the aromatic core as potential antioxidant and anti-cancer drugs. Eur. J. Med. Chem..

[62] Gong Z., Liu X., Dong J., Zhang W., Jiang Y., Zhang J., Feng W., Chen K., Bai J. (2019). Transition from vesicles to nanofibres in the enzymatic self-assemblies of an amphiphilic peptide as an antitumour drug carrier. Nanoscale.

[63] Zhang X., Yin X., Zhang J., Li A., Gong H., Luo Q., Zhang H., Gao Z., Jiang H. (2019). High-resolution mapping of brain vasculature and its impairment in the hippocampus of Alzheimer’s disease mice. Natl. Sci. Rev..

[64] Elsinga P.H., van Waarde A., Paans A.M.J., Dierckx R.A.J.O. (2012). Trends on the Role of PET in Drug Development.

[65] Preshlock S., Tredwell M., Gouverneur V. (2016). ^18^F-Labeling of arenes and heteroarenes for applications in positron emission tomography. Chem. Rev..

[66] Clemente G.S., Zarganes-Tzitzikas T., Dömling A., Elsinga P.H. (2019). Late-stage copper-catalyzed radiofluorination of an arylboronic ester derivative of atorvastatin. Molecules.

[67] Huang H., Banerjee S., Qiu K., Zhang P., Blacque O., Malcomson T., Paterson M.J., Clarkson G.J., Staniforth M., Stavros V.G. (2019). Targeted photoredox catalysis in cancer cells. Nat. Chem..

[68] Novotna B., Vaneková L., Zavřel M., Buděšínský M., Dejmek M., Smola M., Gutten O., Tehrani Z.A., Polidarová M.P., Brázdová A. (2019). Enzymatic preparation of 2’-5’,3’-5’-cyclic dinucleotides, their binding properties to stimulator of interferon genes adaptor protein, and structure/activity correlations. J. Med. Chem..

[69] Landi G., Linciano P., Borsari C., Bertolacini C.P., Moraes C.B., Cordeiro-da-Silva A., Gul S., Witt G., Kuzikov M., Costi M.P. (2019). Structural insights into the development of cycloguanil derivatives as *Trypanosoma brucei* pteridine-reductase-1 inhibitors. ACS Infect. Dis..

[70] Da Silva F., Bret G., Teixeira L., Gonzalez C.F., Rognan D. (2019). Exhaustive repertoire of druggable cavities at protein–protein interfaces of known three-dimensional structure. J. Med. Chem..

[71] Imai Y., Meyer K.J., Iinishi A., Favre-Godal Q., Green R., Manuse S., Caboni M., Mori M., Niles S., Ghiglieri M. (2019). A new antibiotic selectively kills Gram-negative pathogens. Nature.

